# Does Forest Soil Fungal Community Respond to Short-Term Simulated Nitrogen Deposition in Different Forests in Eastern China?

**DOI:** 10.3390/jof9010053

**Published:** 2022-12-29

**Authors:** Zhenyue Liu, Gexi Xu, Di Tian, Quanhong Lin, Suhui Ma, Aijun Xing, Longchao Xu, Haihua Shen, Chengjun Ji, Chengyang Zheng, Xiangping Wang, Jingyun Fang

**Affiliations:** 1 The Key Laboratory for Silviculture and Conservation of Ministry of Education, Beijing Forestry University, Beijing 100083, China; 2 Key Laboratory of Forest Ecology and Environment of National Forestry and Grassland Administration, Ecology and Nature Conservation Institute, Chinese Academy of Forestry, Beijing 100091, China; 3 Key Laboratory for Earth Surface Processes of the Ministry of Education, Institute of Ecology, College of Urban and Environmental Sciences, Peking University, Beijing 100871, China; 4 State Key Laboratory of Vegetation and Environmental Change, Institute of Botany, Chinese Academy of Sciences, Beijing 100093, China

**Keywords:** nitrogen deposition, fungal community, fungal functional groups, forest soil, eastern China

## Abstract

Nitrogen (N) deposition has changed plants and soil microbes remarkably, which deeply alters the structures and functions of terrestrial ecosystems. However, how forest fungal diversity, community compositions, and their potential functions respond to N deposition is still lacking in exploration at a large scale. In this study, we conducted a short-term (4–5 years) experiment of artificial N addition to simulated N deposition in five typical forest ecosystems across eastern China, which includes tropical montane rainforest, subtropical evergreen broadleaved forest, temperate deciduous broadleaved forest, temperate broadleaved and conifer mixed forest, and boreal forest along a latitudinal gradient from tropical to cold temperature zones. Fungal compositions were identified using high-throughput sequencing at the topsoil layer. The results showed that fungal diversity and fungal community compositions among forests varied apparently for both unfertilized and fertilized soils. Generally, soil fungal diversity, communities, and their potential functions responded sluggishly to short-term N addition, whereas the fungal Shannon index was increased in the tropical forest. In addition, environmental heterogeneity explained most of the variation among fungal communities along the latitudinal gradient. Specifically, soil C: N ratio and soil water content were the most important factors driving fungal diversity, whereas mean annual temperature and microbial nutrient limitation mainly shaped fungal community structure and functional compositions. Topsoil fungal communities in eastern forest ecosystems in China were more sensitive to environmental heterogeneity rather than short-term N addition. Our study further emphasized the importance of simultaneously evaluating soil fungal communities in different forest types in response to atmospheric N deposition.

## 1. Introduction

Atmospheric N deposition has become a global environmental issue [1,2]. As a hotspot of global N deposition, forest ecosystems in eastern China are experiencing relatively heavy N fertilization [3,4]. Soil fungi play important roles in accelerating biogeochemical cycles and maintaining the health of soil ecosystems [5]. Changes in soil fungal diversity and community structures may lead to altered nutrient cycling and the multifunctionality of forest ecosystems [6,7]. In forest ecosystems, soil fungal assembly can be functionally classified as symbionts, decomposers, and pathogens [8]. Ectomycorrhizal (EcM) fungi benefit host plants by promoting the acquisition of nutrients and water [9]. Fungal saprotrophs are the most efficient decomposers who are specialized to decay organic matter such as plant litter, dead roots, and root exudates [10]. Pathogenic fungi play critical roles in regulating tree population dynamics and maintenance of plant diversity [11]. Previous studies have reported that N addition reshaped soil fungal community structures and functional compositions [9,12]. For instance, EcM fungal abundance decreased at high levels of N addition in boreal and temperate forests [13]. In a temperate forest, the relative abundance of saprotrophic fungi increased with experimental N addition [12], thus resulting in shifts in community compositions. The abundance of some fungal taxa (e.g., Basidiomycota) and functional groups declined, whereas others (e.g., Ascomycota) increased with the enhancement of N deposition [14,15]. Therefore, knowledge of how soil fungal communities respond to N addition is critical for predicting ecosystem responses and maintaining sustainable forestry under the context of global environmental changes [14].

It is typical that fungi are habitat dependent [16,17], and fungal communities differ among forest types [18]. The effects of N enrichment on soil fungal communities are related to the forest types [19]. N addition experiments in some temperate forests resulted in enhanced soil N availability and thus increased soil fungal richness [15,20], whereas N fertilization in other temperate forests did not change soil fungal richness and diversity [21,22]. By contrast, an increase in fungal diversity was found in short-term N addition experiments in P-limited tropical forests [14]. Moreover, the response of soil fungal community structures to N addition varied across forest ecosystems, and the temperate forests showed a stronger response ratio of community structure than tropical and subtropical forests [19]. Although soil fungal community compositions and diversity generally vary among forest types [8], most of the abovementioned previous results were concluded from experiments in different single ecosystems which limited our further understanding of their variations across various forest types. Therefore, understanding the effects of exogenous N enrichment in different forest types is helpful for predicting the dynamic responses of fungal communities to future environmental changes.

Several recent studies have reported the driving factors of soil fungal diversity and community structure across wide spatial scales, but most studies only focus on climate and soil factors [23,24]. For example, in a global study of soil fungi conducted by Tedersoo et al. [8], the climate was found to be the main environmental factor driving the global distribution of soil fungal diversity. Zhou et al. [25] suggested that mean annual temperature controls forest soil fungal diversity at the continental scale. Bahram et al. [23] proposed that differences in the response of soil fungal diversity to mean annual precipitation led to its global niche differentiation, and the soil C: N ratio was an important parameter for predicting soil fungal richness and biomass. Additionally, a meta-analysis study revealed that N addition decreased soil pH and thus significantly reduced the alpha diversity of soil fungi [26]. Microbial resource limitation quantified by soil extracellular enzyme stoichiometry (EES) was also considered to be an effective way to assess the environmental drivers behind microbial metabolism and ecosystem resource limitation [27,28]. Nevertheless, few studies have linked microbial resource limitation to fungal communities [29]. Moreover, whether previously reported findings were applicable to other ecosystem types remains unclear, especially for different forest ecosystems under the background of global changes.

Hence, we studied fungal diversity, community compositions, and functional differentiation based on 4–5 years of N addition manipulation experiments in five forest ecosystems across eastern China. The forest types in our experimental sites were tropical montane rainforest, subtropical evergreen broadleaf forest, temperate deciduous broadleaf forest, temperate broadleaved and conifer mixed forest, and boreal forest [30]. In these forests, we investigated the characteristics of topsoil fungal diversity, community structures, and functional compositions and explored how soil fungi responded to forest types, short-term N addition, and their interaction. Finally, we partitioned the roles of climate, soil, and microbial nutrient limitations in different forest types as drivers of fungal diversity, community structure, and functional groups. Based on previous studies, we hypothesized that soil fungi in temperate and boreal forests were more sensitive to N deposition than those in subtropical and tropical forests because of the close and slow N cycling in temperate forests and the open and rapid N cycling in tropical forests [19].

## 2. Materials and Methods

### 2.1. Study Sites and Experimental Design

The study was conducted in five forest ecosystems belonging to a project of Nutrient Enrichment Experiments in China’s Forest (NEECF) [30]. Our study was conducted at five sites with distinct forest types along latitudinal span (18°43′ N–50°56′ N, 108°53′ E–129°11′ E, Table 1), which included Jianfengling (JFL), Wuyishan (WYS), Donglingshan (DLS), Wuying (WY), and Genhe (GH). The forest types shift from the tropical montane rainforests in JFL to the boreal coniferous forests in GH [30]. The mean annual temperature (MAT) ranges from −3.4 to 24.7 °C, the mean annual precipitation (MAP) ranges from 487.5 to 2449.0 mm, and the elevation ranges from 350 to 1300 m (Table 1). The nutrient addition experiments in TMF, TBCM, and BF were established in 2010, and those in SEB, and TDB were established in 2011. Three treatments were set up in each forest type: CK (control), LN (50 kg N ha^−1^ year^−1^), and HN (100 kg N ha^−1^ year^−1^), with three replicates of each treatment, for a total of nine sample plots (20 × 20 m^2^; >10 m buffer area). Ammonium nitrate (NH_4_NO_3_) was applied to simulate N deposition in four forests. Meanwhile, urea (CH_4_N_2_O) was applied in DLS due to local safety regulations. More detailed information about the experiment design of NEECF projects can be found in Du et al. [30].

Forest types are compiled from Du et al. [30]. Soil types are classified by USDA soil taxonomy [31]. MAT, mean annual temperature (°C); MAP, mean annual precipitation (mm). JFL: Jianfengling, WYS: Wuyishan, DLS: Donglingshan, WY: Wuying, GH: Genhe. TMF: tropical montane rain forest, SEB: subtropical evergreen broadleaved forest, TDB: temperate deciduous broadleaved forest, TBCM: temperate broadleaved and conifer mixed forest, BF: boreal forest.

### 2.2. Sample Collection

Soil samples were collected in the growing season (July) in 2015, 4–5 years after the start of the fertilizer application. In each plot, we randomly took five soil subsamples (0–10 cm) and mixed them together as one sample. Soil samples were sieved through a 2 mm sterilized mesh after removing litter, roots, stones, and other debris. The mixed soil samples were placed in sterilized sealed bags and transported to the laboratory in a cooler. Each soil sample was divided into two groups [32]. One group was kept at −80 °C for subsequent DNA extraction and fungal sequencing. And the other was divided into four subsamples for the measurements of (1) soil moisture; (2) carbon, nitrogen, phosphorus contents, and pH; (3) microbial biomass; and (4) soil enzyme activities.

### 2.3. Measurements of Soil Physicochemical Properties, Microbial Biomass, and Soil Enzyme Activities

The first subsample was dried at 105 ℃ for 48 h to determine soil moisture. The second subsample was air-dried and then used to determine soil nutrient contents. Soil total C and N concentrations were determined by a CHN analyzer using Dumas combustion (Elementar vario EL III, Elementar, Hanau, Germany). Soil total P concentration was measured by a molybdate/ascorbic acid method after H_2_SO_4_ -HClO_4_ digestion. Soil pH was measured in water suspension with a 1:2.5 soil: water ratio. The third subsample was stored at −4 °C and later used for the determination of microbial biomass C and N by the chloroform fumigation extraction [33]. The extracted MBC and MBN concentrations were determined by a TC/TN analyzer (Multi N/C 3100, Analytik Jena, Jena, Germany). The factors in the transformations from the detected soil total organic carbon and nitrogen to microbial biomass carbon and nitrogen were 0.45 and 0.54, respectively [33].

The fourth subsample for the assays of soil enzyme activities was stored at −20 °C [34]. The activities of β-1,4-glucosidase (BG), cellobiohydrolase (CB), β-1,4-N-acetyl-glucosaminidase (NAG), leucine aminopeptidase (LAP) and acid phosphatase (AP) were measured following the protocol as described by German et al. [35] and Bach et al. [36]. Fresh soil (1.5 g) was suspended in 125 mL sodium acetate buffer (50 mM; pH ¼ 5.3). Soil slurries (200 μL) were added to eight sample assay wells of the 96-well microplate, and 50 μL of 200 μM enzyme substrate was then added and mixed with the soil slurries. Sample control (200 μL soil slurries and 50 μL sodium acetate buffer), quench control (200 μL soil slurries and 50 μL standard substrate (10 μM) of 4-methylumbelliferyl or 7-amino-4-methylcoumarin), and substrate blank (200 μL sodium acetate buffer and 50 μL enzyme substrate) wells were set up in the same way as the sample assay wells. The enzymatic reactions were taken for 2.5 h in the dark at 25 °C. The quantity of fluorescence was measured at 360 nm excitation and 460 nm emission using a microplate reader (Biotek Synergy 2, Winooski, VT, USA). The units for the five enzyme activities were normalized to nmol g^−1^ d.w. soil hour^−1^.

### 2.4. Vector Analysis for Measurement of Microbial Nutrient Limitation

A vector analysis in enzymatic stoichiometry was conducted to measure microbial nutrient limitation [37]. Note that the vector analysis provided measures of the potential and relative resource use limitation rather than the actual resource use limitation for soil microorganisms [38]. In the present study, the vector degree was calculated according to the equations of Jing et al. [38]:

(1) $$Vector\;Degree=atan2\left(y,x\right)\times 180/\pi$$

The x coordinate represents the relative C activity compared to the N-acquiring enzymes [(BG + CB)/(BG + CB + NAG + LAP)], whereas the y coordinate represents the relative C activity compared to the P-acquiring enzymes [(BG + CB)/(BG + CB + AP)] ; *atan2* is a trigonometric function giving the angles in radians between the x-axis and the vector from the plot origin to point (*x*, *y*); 180/*π* is used to convert angles in radians into degree [38]. The vector degree indicates relative microbial P or N limitation (PN_lim), with a value larger than 45° meaning P limitation and a value smaller than 45° meaning N limitation [37].

### 2.5. DNA Extraction, PCR Amplification, and Illumina Sequencing

Soil DNA was extracted using PowerSoil DNA Isolation Kit (MoBio Laboratories, Carlsbad, CA) following the manual. Purity and quality of the genomic DNA were checked on 1% agarose gels and using a NanoDrop spectrophotometer (Thermo Scientific). The ITS1 hypervariable region of the fungal ITS rRNA gene was amplified with the primers ITS1 (5′-CTTGGTCATTTAGAGGAAGTAA-3′) and ITS2 (5′-TGCGTTCTTCATCGATGC-3′). For each soil sample, an 8-digit barcode sequence was added to the 5′ end of the forward and reverse primers (provided by Allwegene Company, Beijing). The PCR was carried out on a Mastercycler Gradient (Eppendorf, Germany) using a 25 μL reaction volume, containing 12.5 μL KAPA 2G Robust Hot Start Ready Mix, 1 µL Forward Primer (5 µM), 1 µL Reverse Primer (5 µM), 5 µL DNA (total template quantity is 30 ng), and 5.5 µL H_2_O. Both forward and reverse primers in the PCR reaction were starting concentration of 5µM, and 1uL of each primer was added to the reaction. Cycling parameters were 95 °C for 5 min, followed by 28 cycles of 95 °C for 45 s, 55 °C for 50 s and 72 °C for 45 s with a final extension at 72 °C for 10 min. The PCR product was purified using an Agencourt AMPure XP Kit. Deep sequencing was performed on the Miseq platform at Allwegene Company (Beijing). After the run, image analysis, base calling, and error estimation were performed using Illumina Analysis Pipeline Version 2.6.

### 2.6. Sequence Data Processing

The raw data was divided into different samples according to the barcode sequence through QIIME [1] (v1.8.0) software. Pear [2] (v0.9.6) software was used to filter and splice raw data. The sequences were removed from consideration if they were shorter than 120 bp, had a low-quality score (≤20), or contained ambiguous bases. After splicing, Vsearch (v2.7.1) software was used to remove sequences with lengths less than 120 bp and removed the chimeric sequence by uchime method according to the Unite Database. Qualified reads were separated using the sample-specific barcode sequences and trimmed with Illumina Analysis Pipeline (v.2.6). Then, the dataset was analyzed using QIIME1 (v1.8.0). Specifically, the sequences were clustered into operational taxonomic units (OTUs) at a similarity level of 97% to generate rarefaction curves. The representative sequence of each OTU was assigned to fungal taxonomic identities by comparison with the UNITE database. Fungal alpha diversity was quantified using Chao1 richness estimator and Shannon diversity index achieved in the software.

The FungalTraits database [10] is a stand-alone spreadsheet dataset presenting a fungal traits and characters database, providing ecological information and functional assignment for environmental research. Based on it, we assigned the fungal guilds according to their primary lifestyles and regard these guilds as hypothetical functional groups. Any OTUs matching with AM fungi were removed. Therefore, the functional groups were grouped into (i) ectomycorrhizal fungi (EcM); (ii) saprotrophs, including soil saprotroph, litter saprotroph, unspecified saprotroph, wood saprotroph, dung saprotroph, nectar/tap saprotroph, and pollen saprotroph; (iii) pathotrophs, including mycoparasite, plant pathogen, animal parasite, algal parasite, lichen parasite, and protistan parasite. For the statistical analyses, we focused only on the functional groups with a relatively high number of OTUs (the ten most abundant functional groups), which are soil saprotroph, ectomycorrhizal, mycoparasite, litter saprotroph, unspecified saprotroph, wood saprotroph, plant pathogen, animal parasite, dung saprotroph, and root endophyte, respectively.

### 2.7. Statistical Analyses

All of our analyses were performed in R (version 4.1.2). We used the quantile comparison plot in the car package to examine the normality of the dependent variable and Bartlett test for homogeneity of variances. Two-way ANOVAs were used to analyze the effects of forest types, N treatments, and their interactions on soil physicochemical properties, soil fungal diversity, the relative abundance of dominant phyla, and dominant functional groups. Variations of soil physicochemical properties, soil fungal diversity, the relative abundance of dominant phyla, and dominant functional groups in each forest type across the treatment were tested using one-way ANOVA followed by the Tukey HSD test. The fungal community structures and functional compositions were visualized by nonmetric multidimensional scaling (NMDS) based on Bray–Curtis distance. In addition, we used the *adonis* function in the vegan package to test the significance of forest types and treatments based on 999 permutations. Transformation-base redundancy analysis (tb-RDA) was used to elucidate the influence of environmental heterogeneity on fungal community structure or functional compositions using the vegan package. To conquer collinearity among predictors, we conducted a variation inflation factor (VIF) analysis and reduced the initial 14 variables (i.e., N treatment, mean annual temperature, mean annual precipitation, soil pH, soil water content (SWC), soil total carbon (STC), soil total nitrogen (STN), soil total phosphorus (STP), the soil C: N ratio, the soil C: P ratio, the soil N: P ratio, microbial biomass carbon (MBC), microbial biomass nitrogen (MBN), and microbial P or N limitation) to 7 variables (i.e., N treatment, mean annual temperature, soil pH, soil water content, the soil C: N ratio, microbial biomass carbon, and microbial P or N limitation). The relative contributions of each environment variable to the fungal diversity, fungal communities, and functional composition were further examined by hierarchical partitioning analyses using the hier.part package [39]. Pearson correlation coefficient was used to test the relationship between environmental variables and fungal diversity indices and the relative abundance of dominant phyla and dominant functional groups, using the psych package.

## 3. Results

### 3.1. Effects of N Addition on Soil Properties and Soil Fungal Diversity

Two-way ANOVAs of forest types and N treatments and their interaction showed that all soil physicochemical properties significantly differed among forest types, whereas N treatments and the interaction of forest types and N treatments had no significant effects on soil properties (Table 2), indicating that differences among forest types are greater than among N treatments in affecting soil physicochemical properties. Specifically, we analyzed the response of soil properties to N addition separately in different forest types (Table S1). The soil C: N ratio was significantly lower under LN and HN treatments compared to that of the CK in the subtropical evergreen broadleaved forest. The soil C:P ratio was significantly lower under the LN treatment compared to that under the HN treatment in the temperate broadleaved and conifer mixed forest.

**Figure 1:**
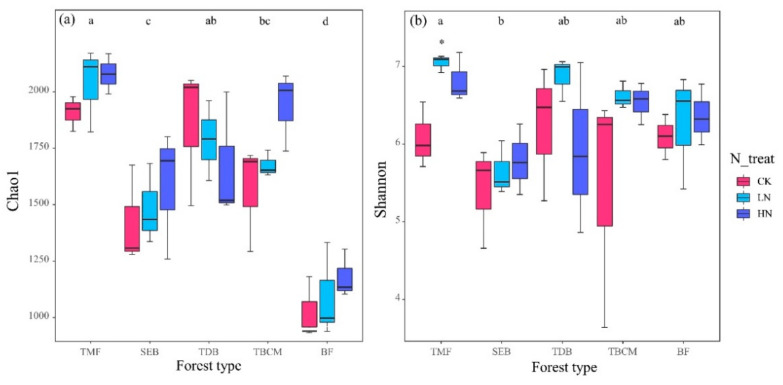
Soil fungal Chao1 index (**a**) and Shannon index (**b**) under N additions in different forest types. Values sharing the same letter are not significantly different among forest types (*p* > 0.05). The stars indicate significant differences in fungal diversity in comparison to the control at *p* < 0.05. TMF: tropical montane rain forest, SEB: subtropical evergreen broadleaved forest, TDB: temperate deciduous broadleaved forest, TBCM: temperate broadleaved and conifer mixed forest, BF: boreal forest.

The Chao1 index and the Shannon index of fungal diversity varied significantly across forest types, whereas the Shannon index differed among N treatments (Table 2). We found that the Chao1 index was the highest in the tropical montane rainforest compared with that in the subtropical evergreen broadleaved forest, the temperate broadleaved and conifer mixed forest, and the boreal forest (Figure 1a). However, there were no significant differences shown on the Chao1 index among the N treatments in the respective forest types (Figure 1a). We found that Shannon diversity in the tropical montane rainforest was significantly higher under LN and HN treatments than that under CK condition (Figure 1b), which was the only significant differentiation shown across the five studied forest types.

### 3.2. Effects of N Addition on Soil Fungal Community Structures, and Functional Compositions

**Figure 2:**
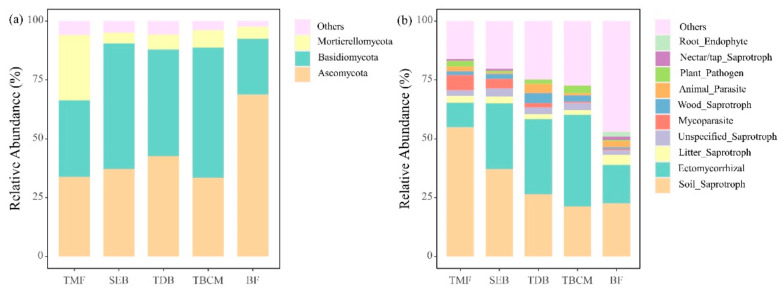
The average relative abundance (%) of fungal taxa at the phylum level (**a**) and the average relative abundance (%) of functional groups (**b**) in control treatments. TMF: tropical montane rain forest, SEB: subtropical evergreen broadleaved forest, TDB: temperate deciduous broadleaved forest, TBCM: temperate broadleaved and conifer mixed forest, BF: boreal forest.

The dominant fungal phyla were Ascomycota (20.10–79.30%), Basidiomycota (6.53–73.88%), and Mortierellomycota (1.49–33.27%), which accounted for more than 90% of the total abundance of fungi in all the forest types (Figure 2a). The relative abundance of Ascomycota was significantly lower in the boreal forest than in other forest types, and the relative abundance of Mortierellomycota was significantly higher in the tropical montane forest than in other forest types (Figure S1). The two-way ANOVAs of forest types and N treatments showed that the three dominant fungal phyla in this study significantly differed among forest types, whereas Basidiomycota significantly differed among N treatments and Mortierellomycota significantly differed among the interaction of forest types and N treatments (Table 3). In the temperate broadleaved and conifer mixed forest, the relative abundance of Basidiomycota was significantly lower under the LN treatment compared to that in the CK. In the tropical montane rainforest, the relative abundance of Mortierellomycota was significantly lower under the LN treatment than in that of the CK.

Among the dominant functional groups, the relative abundances of soil saprotroph and mycoparasite decreased from tropical to boreal forest, respectively (Figure 2b). However, the relative abundance of EcM fungi was higher in temperate and boreal forests than those in the tropical forests (Figure S2), and the relative abundance of litter saprotroph was highest in the boreal forest (Figure 2b). Our two-way ANOVAs of forest types and N treatments showed that 8 of 10 fungal functional groups significantly differed between forest types, whereas only 2 of 10 functional groups differed among N treatments, indicating that differences among forest types were stronger than differences among treatments in affecting fungal functional compositions (Table 3). The relative abundance of soil saprotrophic fungi decreased under N treatments in the subtropical evergreen broadleaved forest (Table S3). In the temperate broadleaved and conifer mixed forest, the relative abundance of mycoparasite was significantly higher under the LN treatment than that under the CK condition. However, other functional groups showed no significant responses to short-term N addition treatment.

**Figure 3:**
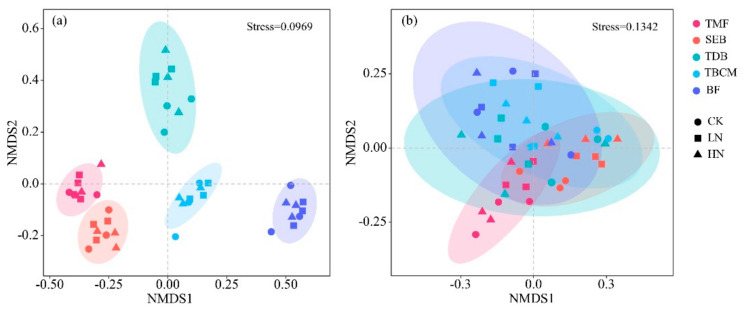
Nonmetric multidimensional scaling (NMDS) ordination plot based on the Bray–Curtis distance of samples for the fungal community structures (**a**) and fungal functional groups (**b**) in five forest types under different N treatments. TMF: tropical montane rain forest, SEB: subtropical evergreen broadleaved forest, TDB: temperate deciduous broadleaved forest, TBCM: temperate broadleaved and conifer mixed forest, BF: boreal forest.

NMDS analysis revealed significant differences in fungal community structure across forest types (Figure 3a). The PERMANOVA test showed that soil fungal community structure varied significantly across different forest types (Table S4 and Table S6) rather than across different N treatments (Table S4 and Table S7). Moreover, soil fungal functional compositions also varied significantly across different forest types (Figure 3b, Table S5) rather than across different N treatments (Table S5 and Table S9). The functional compositions of the two temperate forests (TDB and TBCM) were similar (Table S8, *p* = 0.097), but there were significant differences between the remaining forest types (*p* < 0.05). Overall, N addition showed weak effects on soil fungal community structures and functional composition (Tables S4 and S5).

### 3.3. Relationships between Fungal Communities and Environment Variables

**Figure 4:**
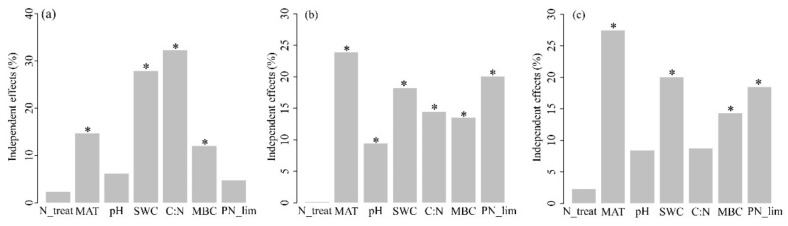
The relative influence of environmental factors on the fungal alpha diversity (**a**), fungal community structures (**b**), and fungal functional compositions (**c**) revealed by hierarchical partitioning analysis. N_treat: N treatments, MAT: mean annual temperature, pH: soil pH, SWC: soil water content, C:N:the soil C: N ratio, MBC: microbial biomass carbon, PN_lim: microbial P or N limitation. Level of significance: * *p* < 0.05.

The results of hierarchical partitioning analyses (Figure 4) showed that soil C: N ratio, SWC, MAT, and MBC explained 31.8%, 26.4%, 12.8%, and 11.0% of soil fungal alpha diversity variance, respectively. Soil fungal community structures were significantly affected by MAT, PN_lim, SWC, soil C:N ratio, MBC, and pH with explanations of 23.9%, 20.1%, 18.2%, 14.5%, 13.6%, and 9.5%, respectively. Soil fungal functional compositions were significantly affected by MAT, SWC, PN_lim, and MBC with explanations of 27.5%, 20.0%, 18.5%, and 14.4%, respectively.

**Figure 5:**
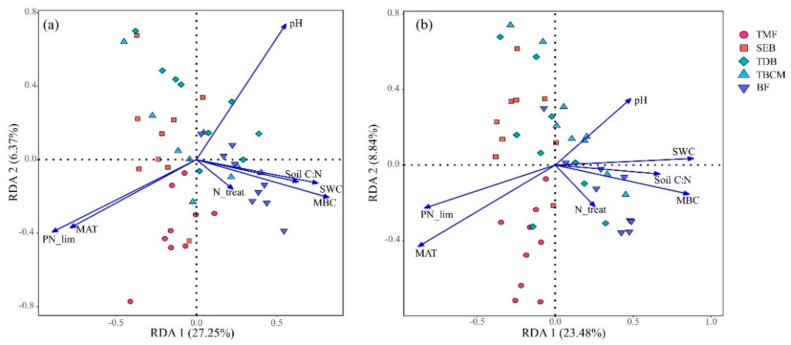
The results of redundancy analysis (RDA) showed the correlations between the fungal community and soil environmental heterogeneity across different forest types (*n* = 9), for fungal phylum-level taxonomy (**a**) and functional compositions (**b**). TMF: tropical montane rain forest, SEB: subtropical evergreen broadleaved forest, TDB: temperate deciduous broadleaved forest, TBCM: temperate broadleaved and conifer mixed forest, BF: boreal forest.

Across the first two canonical axes, the RDA explained 32.62% and 32.32% of the relationship between fungi taxonomic and functional groups, respectively (Figure 5). The results showed that mean annual temperature and PN_lim were two important environmental factors shaping soil fungal community structures and their functional compositions among N treatments and across forest types. Furthermore, soil pH was in the opposite direction of the above two factors. The change in pH was also significantly related to the changes in fungal community structure and fungal functional groups.

## 4. Discussion

### 4.1. Unsensitivity of Forest Soil Fungi Respond to Short-Term N Deposition

Our study explored fungal diversity, community structure, and functional compositions and their responses to short-term N deposition in five typical forests in five forest types in eastern China. Exogenous N input to forest ecosystems might change soil fungal diversity and community both directly (soil-mediated) and indirectly (plant-mediated) [19,26]. N addition was generally believed to decrease soil pH [26]. By contrast, in our study, no differences were found statistically in soil pH under N treatment from tropical to boreal forests. This pattern may be attributed to short-term N additions which may not be strong enough to break up the buffer range of soil pH [40]. Moreover, there were no significant changes of soil total N among N treatments in each forest type, which indicated that the additional N had possibly been taken up by plants [3].

Impacts of N deposition on soil fungal communities depend on forest types which are structured by climate change and environmental heterogeneity [19]. We found that N addition significantly increased fungal Shannon diversity in tropical rainforests (Figure 1). This pattern may be caused by N addition reducing the microbial P limitation in the tropical montane rainforest (Table S1) which may promote fungal growth. On the other hand, N addition enhanced the plant’s net primary productivity [41], which in turn supplies more resources for soil fungal growth [42]. We expected that the influence of N addition would be more significant in the boreal and temperate forests than those in the tropical and subtropical forests. In contrast to our hypothesis, soil fungi in the tropical forest in this study may be more sensitive to short-term N additions, whereas there was no statistical difference for soil fungal alpha diversity to short-term N additions in subtropical, temperate, and boreal forests (Figure 1). It has been suggested that N addition experiments in N-limited ecosystems increased soil N availability and thus enhanced soil fungal diversity [15,20,43]. However, the calculation of microbial nutrient limitation showed that only boreal forest reflected weak N limitation (PN_lim slightly less than 45), whereas the other forest types showed stronger P limitation [37]. Therefore, no evidence of an increase of fungal diversity in response to N addition due to N-limitation was detected in this study. Many studies have concluded that N addition has no significant effect on fungal richness and diversity indices [21,22,27,44]. A global meta-analysis also found that the effect of short-term (≤5 years) N addition on the fungal Chao1 index was not significant, whereas the index decreased significantly when N addition over five years [45]. Therefore, we believe that the insensitivity of soil fungal alpha diversity response to N addition in this study may be due to the weak microbial N limitation at the experimental forests and the short duration of the N addition.

NMDS analysis based on Bray–Curtis distance showed that the fungal community structures did not change significantly during the short-term N addition, whereas they were significantly different among forest types (Figure 3). Short-term N addition changed the relative abundance of some dominant fungal phyla. In the temperate broadleaved and conifer mixed forest, the relative abundance of fungi in Basidiomycota decreased with increasing N addition and soil nutrient enrichment (Table S2), resulting in the competitive pressures on fungi in Ascomycota for resources reduced [15]. Many previous studies have obtained the same conclusions [14,15,43]. In addition, Wang et al. [46] found the relative abundance of Ascomycota also tended to decrease with N addition even though there were no changes in the relative abundance of Basidiomycota. In our study, the relative abundance of fungi in Mortierellomycota (represented only by the genus *Mortierella* [47]) decreased significantly after N addition in the tropical rainforest (Table S2). Several studies have shown that *Mortierella* sp. could defend against soil degradation, improve soil health, stimulate the production of plants, and have the functional ability to degrade a range of toxic organic compounds [6]. Therefore, we speculate that the decrease in the relative abundance of Mortierellomycota caused by N deposition may have a negative impact on tropical forests. The apparent discrepancies in response to N addition across forest types are not entirely surprising due to the large variations in the climate of research areas and soil nutrient conditions.

For fungal functional compositions, we found significant differences in functional groups across forest types with the exceptions in the two temperate forests (Figure 3b, Table S8). Previous studies have noted that litter in mixed coniferous and broadleaf forests leads to a similar microbial community structure among different tree species [48], which may explain the similar fungal functional compositions in these two temperate forests. In the temperate deciduous broadleaved forest, the soil C: N ratio decreased and the relative abundance of soil saprotrophic fungi under LN and HN treatments was significantly lower than those under CK treatment, which may be due to the direct toxic effect of high levels of N deposition on fungal saprotrophs [49]. Furthermore, soil N inputs appear to promote the growth and competitive ability of N-tolerant, copiotrophic taxa, which may also have generally lower capacities for organic matter degradation [50]. A decline in the relative abundance of fungal saprotrophs following N addition was offset by an increase in the relative abundance of EcM fungi [12]. However, some studies have also suggested that N deposition increased the relative abundance of saprotrophic fungi and decreased the relative abundance of EcM fungi [14,51]. These phenomena may reflect the competition for limited organic resources between EcM and saprotrophic fungi, which can be supported by the “Gadgil effect” [52]. Such shifts in fungal functional composition could affect soil enzymatic activities and litter decomposition, thereby regulating soil biogeochemical cycling in forests exposed to atmospheric N deposition [53].

### 4.2. Factors Shaping Soil Fungal Community in Chinese Eastern Forests

To predict the responses of ecosystem functions to potential global environmental changes, it is necessary to understand how environmental factors shape fungal communities [46]. We explored the environmental drivers of soil fungal communities in forests under N deposition in five forest types. Specifically, soil C: N ratio and soil water content were the most important factors affecting fungal diversity (Figure 4a). Mean annual temperature and microbial nutrient limitation were the key variables driving the variations in fungal community structures and functional compositions (Figure 4b,c). We originally expected that N addition would alleviate N-limitation and shift soil fungal community in boreal and temperate forests. In fact, the results of our study showed that N addition did not affect soil fungal diversity, community structures, and functional compositions in most cases. The duration of N addition (4 or 5 years) may be a major explanatory factor that leads to these unexpected non-significant responses of soil fungi to N addition in temperate and boreal forests. Numerous studies reported that long-term fungal responses may be more significant than short-term fungal responses to N addition. Previous studies have suggested that changes in fungal diversity and community structures increased significantly as the N addition study duration increased [19,45]. However, another global meta-study came to a different conclusion, with a decrease in the fungal Chao1 index for N application in less than ten years, but no significant change for 10–20 years [26]. A study in tropical forests found that soil fungi were more sensitive under short-term N addition than long-term N addition [14]. This may support our findings that tropical forest soil fungi are more sensitive to short-term N additions, and the effects of the duration of N addition on soil fungal communities in these forest ecosystems require further study.

The most important environmental factors driving soil fungal alpha diversity were soil C: N ratio and soil water content, which were the same as results of Ni et al. [54] and Liu et al. [55], indicating that fungal diversity changes depend mostly on soil nutrient supply. Because changes in soil C: N ratio can substantially influence the fungal anabolism and foraging strategies [54]. In this study, the fungal Chao1 index was negatively correlated with the soil C: N ratio (Figure S3a). Higher soil C: N ratio may break the stoichiometric balance between soil and mycelia, restraining the activity of exoenzymes and the accumulation of fungal biomass [56]. Therefore, the highest soil C: N ratio formed the lowest Chao1 index of soil fungi in this study. In addition, the soil C: N ratio will also co-vary with the community composition of aboveground plants [54]. Consequently, the observed pattern of fungal diversity driven by the soil C: N ratio in this study may be partly attributed to the variation of aboveground plant communities. Soil water content also played an important role in shaping soil fungal alpha diversity in forest ecosystems. In this study, it was observed that soil water content showed a significant negative correlation with the fungal Chao1 index (Figure S3). Similarly, the effect of SWC on fungal diversity was also reported in other forest ecosystems, such as the forests in western Canada [57] and eastern China [58,59] This may be because soil water content influences the O_2_ level in the soil and affects soil nutrient availability and transportation [29], which in turn structures soil fungal metabolic pathways and community composition [60].

In our study, mean annual temperature and PN_lim were the strongest predictor of fungal community structures and functional compositions in the topsoil of five forest types (Figure 4 and Figure 5). Several global-scale studies have confirmed that temperature determined the distribution of soil fungal communities [8,61]. Moreover, studies on the soil fungal community structure of forests in eastern China have also obtained the same conclusions [62,63]. In addition, MAT was positively correlated with the relative abundances of three fungal dominant phyla, soil saprotroph, and mycoparasite (Figure S3). A recent study found that the physiological features of saprotrophic fungi are sensitive to temperature, and temperature thresholds control the global distribution of fungal decomposers [64]. Moreover, temperature affects fast-growing opportunistic fungi (pathogenic fungi). Delgado-Baquerizo et al. [11] found that warmer temperatures increased the relative abundance of soil-borne potential plant fungal pathogens. Temperature accelerates metabolic rates and biochemical processes directly, linking abiotic environmental changes to fungal communities and thus affecting their growth [25]. Meanwhile, our results suggested that fungal community structures and functional compositions varied according to PN_lim (Figure 4 and Figure 5). The activity of soil extracellular enzymes links environmental nutrient availability with microbial metabolic requirements [28]. In this study, correlation analysis showed that the relative abundance of three dominant fungal phyla (Ascomycota, Basidiomycota, and Mortierellomycota) had significant correlations with PN_lim. Cui et al. [29] found similar patterns in agroecosystems that the stronger the N limitation, the higher the relative abundance of Ascomycota. However, fungal abundance in Basidiomycota displayed the opposite pattern. Both patterns demonstrate that N limitation influence soil fungal community structures significantly. Microbial community structures and functional compositions determine microbial community function and metabolic patterns [65]. Microbial resource limitation favors fungal groups with different resource preferences, and trophic strategies may support the different responses of soil fungi to resource imbalances [29]. In addition, the soil extracellular enzyme stoichiometry theory, which characterizes the relationship between microbial element requirements and resources [28], has been widely used to explore nutrient cycling in terrestrial ecosystems [66]. This study further verifies that it is still feasible to explain the topsoil fungal community structures and functional compositions of forest ecosystems by microbial nutrient limitation calculated by EES at a large scale. An in-depth study of the impact of microbial resource limitation on fungal community structures and functions may help predict the dynamics and nutrient cycling in forest ecosystems under the background of global climate change.

## 5. Conclusions

There were significant differences in soil fungal diversity, community structures, and functional compositions across different forest types in eastern China. This study demonstrated that continued N deposition for 4–5 years only resulted in an increase in the fungal diversity in the tropical montane rainforest. In addition, the relative abundance of some specific fungi taxa (e.g., Mortierellomycota in tropical montane forest, Basidiomycota in temperate broadleaved and conifer mixed forest) and fungal functional groups (e.g., soil saprotroph in the subtropical evergreen forest) showed a decrease in short-term N addition experiments. However, short-term N additions overall showed weak effects on topsoil fungal diversity, community structures, and functional compositions in forests of eastern China. Soil C:N ratio and soil water content were the most important factors affecting fungal alpha diversity. Mean annual temperature and microbial nutrient limitation (PN_lim) were the key variables driving variations of fungal community structures and functional compositions across different forest types. Our results enhance the understanding of the responses of soil fungi to N deposition in typical forests across eastern China, which will shed light on biogeochemical cycling in forest ecosystems under global climate change.

## Figures and Tables

**Table 1:** General information on the NEECF sites.

Biome	Site	Forest Type	Soil Type	Location	Altitude	MAT	MAP
					(m)	(°C)	(mm)
Tropical forest	JFL	TMF	Inceptisols	18°43′ N, 108°53′ E	870	24.7	2449
Subtropical forest	WYS	SEB	Ultisols	27°39′ N, 117°57′ E	700	18.6	2159.4
Temperate forest	DLS	TDB	Alfisols	39°58′ N, 115°26′ E	1300	9.8	472.1
	WY	TBCM	Alfisols	48°07′ N, 129°11′ E	350	1.8	719.4
Boreal forest	GH	BF	Alfisols	50°56′ N, 121°30′ E	825	−3.4	487.5

**Table 2:** Effects of forest types, N treatments, and their interaction on variables (i.e., soil properties and fugal diversity) based on two-way analyses of variance (ANOVAs). Level of significance: * *p* < 0.05, ** *p* < 0.001.

Variables	F-Values		
	Forest Type	N Treat	Forest Type × N Treat
pH	166.87 **	2.01	1.94
SWC	112.85 **	0.62	0.99
STC	715.45 **	0.85	1.33
STN	203.31 **	1.18	0.77
STP	95.94 **	0.41	0.33
C:N	56.57 **	3.2	1.4
C:P	163.18 **	0.59	1.6
N:P	75.72 **	0.35	1
MBC	23.91 **	0.24	1.04
MBN	20.05 **	0.17	0.58
PN_lim	120.27 **	0.7	0.59
Chao1	27.07 **	1.74	0.82
Shannon	2.97 *	3.73 *	0.78

pH: soil pH, SWC: soil water content, STC: soil total carbon, STN: soil total nitrogen, STP: soil total phosphorus, C:N:the soil C:N ratio, C:P:the soil C:P ratio, N:P:the soil N:P ratio, MBC: microbial biomass carbon, MBN: microbial biomass nitrogen, PN_lim: microbial P or N limitation.

**Table 3:** Effects of forest types, N treatments, and their interactions on variables (i.e., the relative abundance of dominant fungal phyla and functional groups) based on two-way analyses of variance (ANOVAs). Level of significance: * *p* < 0.05, ** *p* < 0.001.

Variables	F-Values		
	Forest Type	N Treat	Forest Type × N Treat
Ascomycota	9.72 **	3.18	0.88
Basidiomycota	5.98 **	3.37 *	1.3
Mortierellomycota	16.7 **	0.13	2.86 *
Soil_saprotroph	14.14 **	3.68 *	1.76
Ectomycorrhizal	5.98 **	1.32	1.54
Mycoparasite	16.17 **	3.27	0.95
Litter_saprotroph	2.16	0.51	0.17
Unspecified_saprotroph	3.06 *	1.26	1.86
Wood_saprotroph	7.96 **	4.93 *	1.6
Plant_pathogen	11.27 **	2.67	1
Animal_parasite	8.51 **	0.2	0.46
Dung_saprotroph	1.74	3.1	0.78
Root_endophyte	25.13 **	0.63	2.14

## Data Availability

We have deposited the sequencing data in NCBI with accession number PRJNA909592.

## Supplementary Materials

The following supporting information can be downloaded at: https://www.mdpi.com/article/10.3390/jof9010053/s1, Figure S1: the relative abundance of dominant fungal phyla under N additions in different forest types, Figure S2: the relative abundance of dominant fungal functional guilds under N additions in different forest types, Figure S3: correlations between environmental factors and fungal α-diversity (a), dominant phyla (b), and dominant functional groups (c) under all N treatments, Table S1: effects of N treatment on the soil physicochemical properties, microbial biomass, and microbial resource limitation, Table S2: effects of N treatment on the relative abundance of the top three dominant fungal phyla, Table S3: effects of N treatment on relative abundance of the top ten dominant fungal functional groups, Table S4: results of Adonis tests for effects of forest types, N treatments, and their interactions on soil fungal communities, Table S5: results of Adonis tests for effects of forest types, N treatments, and their interactions on soil fungal functional compositions, Table S6: results of Adonis tests comparing pair-wise fungal community similarities derived in the matrix (Bray–Curtis distance) for each forest type and examining the significance of separation in different forest types, Table S7: results of Adonis tests comparing pair-wise fungal community similarities derived in the matrix (Bray–Curtis distance) for each N treatment and examining the significance of separation in different N treatments, Table S8: results of Adonis tests comparing pair-wise fungal functional composition similarities derived in the matrix (Bray–Curtis distance) for each forest type and examining the significance of separation in different forest types; Table S9. results of Adonis tests comparing pair-wise fungal functional composition similarities derived in the matrix (Bray–Curtis distance) for each N treatment and examining the significance of separation in different N treatments.
