# Supplementary material for: Does Forest Soil Fungal Community Respond to Short-Term Simulated Nitrogen Deposition in Different Forests in Eastern China?
Source: J Fungi (Basel). 2022 Dec 29;9(1):53. doi: 10.3390/jof9010053 (PMC9864950; doi:10.3390/jof9010053)
Supplement: Supplementary file 1 [file jof-09-00053-s001.zip › jof-1978382-supplementary.pdf]

Supplementary material

Does Forest Soil Fungal Community Respond to Short-Term Simulated Nitrogen Deposition in Eastern China?

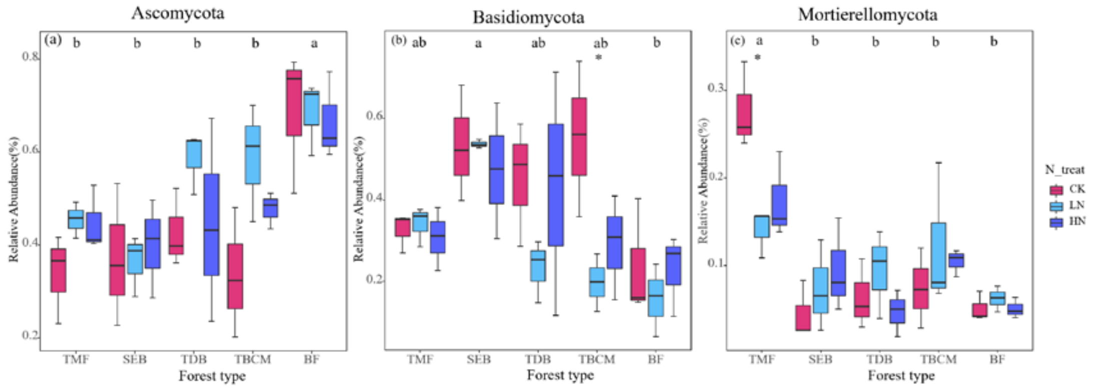

Figure S1. The relative abundance of dominant fungal phyla under N additions in different forest types. Values sharing the same letter are not significantly different among forest types (P>0.05). The stars indicate significant differences in fungal diversity in comparison to the control at P < 0.05. TMF: tropical montane rain forest, SEB: subtropical evergreen broadleaved forest, TDB: temperate deciduous broadleaved forest, TBCM: temperate broadleaved & conifer mixed forest, BF: boreal forest.

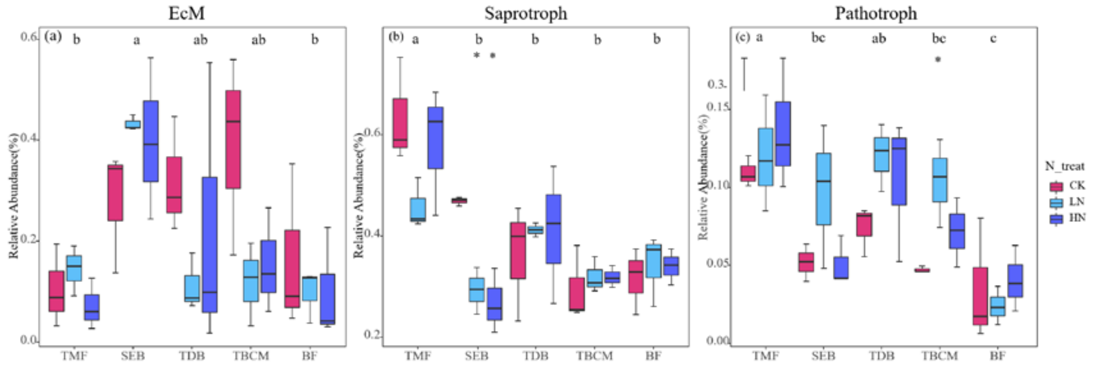

Figure S2. The relative abundance of dominant fungal functional guilds under N additions in different forest types. Values sharing the same letter are not significantly different among forest types (P > 0.05). The stars indicate significant differences in fungal diversity in comparison to the control at P < 0.05. TMF: tropical montane rain forest, SEB: subtropical evergreen broadleaved forest, TDB: temperate deciduous broadleaved forest, TBCM: temperate broadleaved & conifer mixed forest, BF: boreal forest.

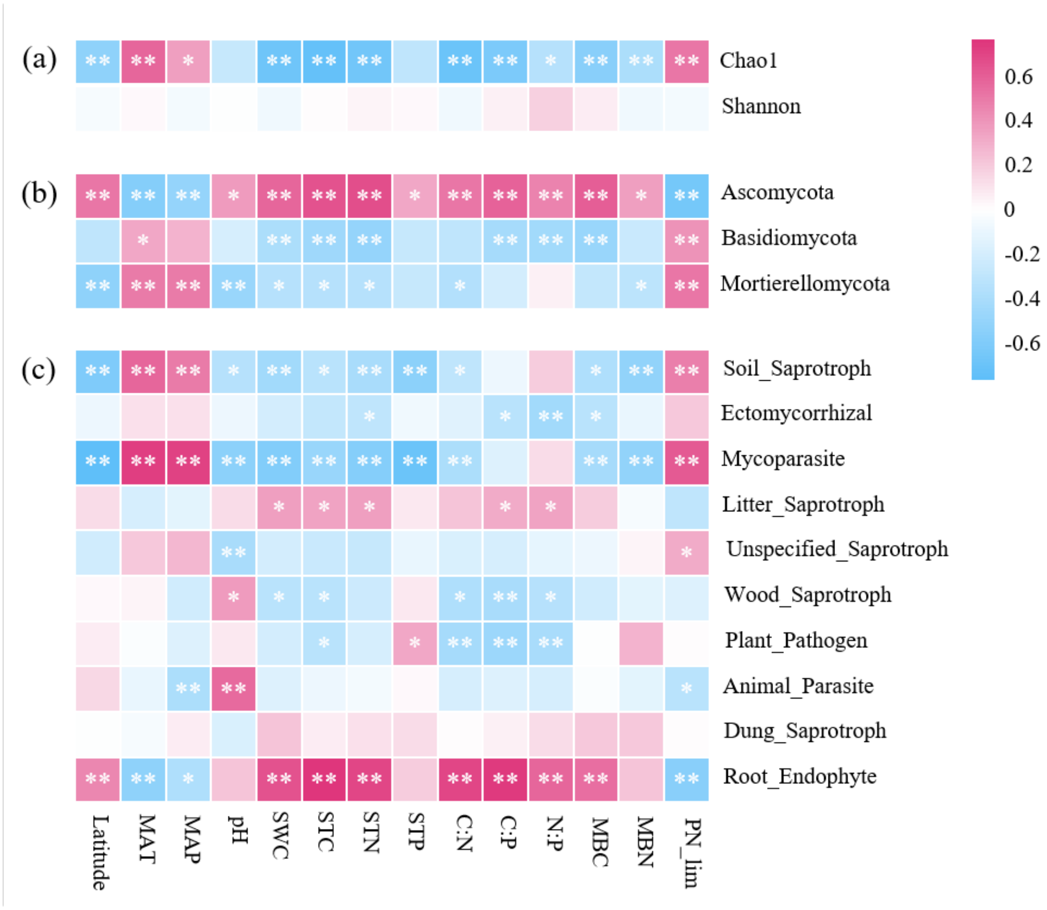

Figure S3. Correlations between environmental factors and fungal α-diversity (a), dominant phyla (b), and dominant functional groups (c) under all N treatments. Dominant phyla and dominant functional groups refer to the relative abundance of the top three phyla and the top ten functional groups, respectively. The correlation was evaluated by the Pearson correlation coefficient. Blue indicates a negative correlation, and pink is a positive correlation, and the transparency of color reflects the strength of the correlation. Level of significance: * $P < 0.05$, ** $P < 0.001$. MAT: mean annual temperature (°C), MAP: mean annual precipitation (mm), pH: soil pH, SWC: soil water content, STC: soil total carbon, STN: soil total nitrogen, STP: soil total phosphorus, C:N: the soil C: N ratio, C:P: the soil C: P ratio, N:P: the soil N: P ratio, MBC: microbial biomass carbon, MBN: microbial biomass nitrogen, PN_lim: microbial P or N limitation.

Table S1. Effects of N treatment on the soil physicochemical properties, microbial biomass, and microbial resource limitation. Data were presented as means ± standard errors (n=3), and the different letters within columns indicate significant differences among treatments in the same forest type ($P < 0.05$). pH: soil pH, SWC: soil water content, STC: soil total carbon, STN: soil total nitrogen, STP: soil total phosphorus, C:N: the soil C: N ratio, C:P: the soil C: P ratio, N:P: the soil N: P ratio, MBC: microbial biomass carbon, MBN: microbial biomass nitrogen, PN_lim: microbial P or N limitation. TMF: tropical montane rain forest, SEB: subtropical evergreen broadleaved forest, TDB: temperate deciduous broadleaved forest, TBCM: temperate broadleaved & conifer mixed forest, BF: boreal forest.

Forest type	Treat	pH	SWC	STC (mg g^{-1})	STN	STP	C:N	C:P	N:P	MBC (mg kg^{-1})	MBN	PN_lim
TMF	CK	4.4±0.1a	20±2a	18.3±2.6a	1.5±0.2a	0.1±0a	11.8±0a	147.7±14.7a	12.5±1.2a	368.6±41.1a	44.8±4.5a	**88.2±0.3a**
	LN	4.3±0.1a	17.9±2.4a	21.7±4a	1.7±0.3a	0.1±0a	12.6±0.2a	169.3±20.4a	13.5±1.9a	678.5±167.1a	49.6±10.2a	**87.2±0.1a**
	HN	4.3±0.1a	19.7±4a	22.9±5.2a	1.9±0.4a	0.2±0a	11.9±0.3a	150.2±10.5a	12.6±1a	465.5±59.4a	28.2±8.1a	**85.0±0.9b**
SEB	CK	4.6±0.1a	26.7±2.2a	36.4±6.9a	2.3±0.4a	0.3±0a	**15.4±0.5a**	135±19a	8.7±1a	512.6±4.9a	109.5±10.4a	80.9±1.2a
	LN	4.5±0a	26.4±0.3a	29.6±1.9a	2.1±0.1a	0.3±0a	**13.8±0.3b**	99.6±6.1a	7.2±0.3a	427.3±55.3a	90.5±10.2a	78.9±4.4a
	HN	4.5±0a	26.1±1.1a	29.3±1.3a	2.1±0.1a	0.3±0a	**13.8±0.2b**	99±3.2a	7.2±0.1a	278.3±91.1a	82.2±18.5a	80±1.5a
TDB	CK	6.5±0.2a	22.2±0.2a	37.1±5.8a	3.1±0.5a	0.6±0.1a	12±0.2a	67.2±5.4a	5.6±0.4a	544.9±75.4a	73.7±7.4a	57.4±4.6a
	LN	7±0.1a	20.8±0.3a	48.8±7.1a	4.1±0.5a	0.5±0a	11.9±0.3a	91.8±16.7a	7.7±1.2a	658.6±174.8a	95±21.2a	52.4±4.7a
	HN	6.9±0.1a	20.9±1.8a	36.2±5.7a	3.2±0.6a	0.5±0.1a	11.3±0.2a	67.2±3a	6±0.2a	528.2±70.4a	69.1±7.3a	58.6±2.9a
TBCM	CK	5.5±0.1a	41±3.3a	104.8±1.3a	6±1a	1.2±0.1a	18.9±4a	**87±8.2ab**	4.8±0.6a	971.7±91.4a	219.3±22.9a	63.4±2.7a
	LN	5.3±0.1a	42±2a	79.3±14.1a	6±1.1a	1.2±0.2a	13.1±0.4a	**64.5±9b**	4.9±0.5a	1355.2±524.9a	281.8±100.8a	63.3±1.3a
	HN	5.1±0.1a	42.9±0.3a	108.2±6.3a	7.2±0.5a	1.1±0.1a	15.1±0.2a	**98.9±4.4a**	6.5±0.3a	1149.8±268.4a	235.7±41.2a	62.4±1.8a
BF	CK	5.9±0.1a	64.4±3.1a	323.2±17.3a	12.7±0.9a	0.8±0a	25.5±0.5a	398.4±23.3a	15.7±1.2a	1942.6±415.4a	191.3±29.2a	43.5±2.6a
	LN	6.1±0.1a	67.5±1a	325.7±14.9a	14.4±0.4a	0.9±0a	22.6±0.5a	368.2±32.2a	16.2±1.2a	1607.2±427.3a	184.3±51.3a	43.2±1.1a
	HN	5.6±0.3a	55.8±8.4a	343.2±9a	13.4±0.8a	0.8±0.1a	25.8±2.1a	440.1±43.4a	17±0.5a	2406.5±158.6a	250.6±20a	48.2±3.8a

Table S2. Effects of N treatment on the relative abundance of the top three dominant fungal phyla. Data were presented as means ± standard errors (n=3), and the different letters within columns indicate significant differences among treatments in the same forest type ($P < 0.05$). TMF: tropical montane rain forest, SEB: subtropical evergreen broadleaved forest, TDB: temperate deciduous broadleaved forest, TBCM: temperate broadleaved & conifer mixed forest, BF: boreal forest.

Forest type	Treat	Ascomycota	Basidiomycota	Mortierellomycota
TMF	CK	33.75±5.54a	32.6±2.82a	**27.69±2.84a**
	LN	45.42±2.23a	34.06±2.8a	**14.04±1.59b**
	HN	44.68±4.05a	30.69±4.45a	**17.39±2.83ab**
SEB	CK	37.12±8.83a	53.37±8.17a	4.49±1.9a
	LN	36.32±3.79a	53.69±0.61a	7.35±3a
	HN	39.83±6.14a	47.27±9.59a	9.51±3.09a
TDB	CK	42.66±4.85a	45.25±8.81a	6.34±2.3a
	LN	58.6±3.89a	23.26±4.45a	9.42±2.91a
	HN	44.66±12.64a	42.95±17.27a	4.69±1.53a
TBCM	CK	33.45±8.05a	**55.26±10.96a**	7.38±2.64a
	LN	58.72±7.33a	**19.8±4.04b**	12.18±4.78a
	HN	47.67±2.25a	**29.1±7.39ab**	10.43±0.89a
BF	CK	68.72±8.87a	23.71±8.3ab	5.13±0.98a
	LN	68.41±4.62a	15.73±5.11a	6.22±0.83a
	HN	66.57±5.43a	22.84±5.79a	5.06±0.67a

Table S3. Effects of N treatment on the relative abundance of the top ten dominant fungal functional groups. Data were presented as means ± standard errors (n=3), and the different letters within columns indicate significant differences among treatments in the same forest type (*P* < 0.05). TMF: tropical montane rain forest, SEB: subtropical evergreen broadleaved forest, TDB: temperate deciduous broadleaved forest, TBCM: temperate broadleaved & conifer mixed forest, BF: boreal forest.

Forest type	Treat	Soil_saprotroph	Ectomycorrhizal	Mycoparasite	Litter_saprotroph	Unspecified_saprotroph	Wood_saprotroph	Plant_pathogen	Animal_parasite	Dung_saprotroph	Root_endophyte
TMF	CK	54.79±6.77a	10.48±4.73a	6.32±1.28a	2.88±0.0a	2.47±0.22a	1.71±0.44a	2.59±0.66a	1.88±1.15a	0.73±0.29a	0.04±0.02a
	LN	34.69±2.68a	14.44±2.86a	8.64±2.42a	3.06±0.56a	3.08±0.25a	3.03±0.49a	2.45±0.15a	0.71±0.2a	1.54±0.58a	0.1±0.04a
	HN	44.84±7.9a	7.12±2.91a	8.28±1.12a	4.32±0.2a	5.61±1.64a	2.69±1.08a	3.48±1.33a	1.36±0.53a	0.67±0.13a	0.07±0.03a
SEB	CK	**37.03±1.43a**	27.95±7.13a	3.93±0.41a	2.9±0.76a	3.53±0.66a	2.27±0.72a	0.91±0.36a	0.35±0.05a	0.36±0.27a	0.18±0.05a
	LN	**20.51±2.9b**	43.23±0.88a	7.96±2.86a	2.22±0.35a	4.07±1.45a	1.93±0.44a	1.21±0.15a	0.54±0.09a	0.23±0.07a	0.13±0.05a
	HN	**19.44±2.52b**	39.95±9.22a	3.05±0.34a	2.62±0.54a	2.11±0.11a	1.76±0.6a	1.12±0.27a	0.88±0.33a	0.59±0.18a	0.39±0.22a
TDB	CK	26.4±5.85a	31.93±6.58a	1.69±0.09a	2.17±1.24a	2.89±0.78a	4.32±2.94a	1.84±0.5a	3.86±0.64a	0.19±0.07a	0.09±0.03a
	LN	26.6±2.19a	11.21±3.23a	3.02±0.68a	2.93±0.69a	1.54±0.24a	9.48±1.07a	4.58±1.54a	4.32±0.7a	0.23±0.12a	0.53±0.28a
	HN	31.11±7.5a	22.35±16.65a	2.68±1.58a	4.05±2.76a	2.18±0.43a	3.31±0.59a	3.66±1.67a	4.16±1.07a	0.1±0.04a	0.05±0.02a
TBCM	CK	21.23±3.83a	38.97±11.43a	**0.53±0.13b**	1.98±0.17a	3.05±0.19a	2.64±0.87a	3.29±0.4a	0.89±0.34a	0.5±0.27a	0.05±0.02a
	LN	19.84±4.3a	11.85±4.74a	**2.05±0.51a**	2.53±0.74a	3.79±0.9a	4.12±0.44a	6.92±1.5a	1.42±0.22a	1.54±0.76a	0.03±0.01a
	HN	21.84±0.57a	15.43±5.99a	**1.16±0.2ab**	1.84±0.15a	4.57±1.22a	2.74±0.35a	4.81±1.5a	1.19±0.38a	0.5±0.33a	0.06±0.01a
BF	CK	22.54±2.75a	16.36±9.56a	0.41±0.04a	4.37±1.11a	1.99±0.27a	0.77±0.38a	0.53±0.3a	2.5±2.37a	0.52±0.3a	2.05±0.59a
	LN	22.89±4.02a	9.84±3.06a	0.98±0.29a	5.66±2.53a	1.68±0.5a	2.02±1.68a	0.53±0.08a	0.87±0.36a	1.75±1.28a	0.9±0.43a
	HN	22.94±1.94a	9.99±6.38a	2.26±1.19a	6.24±3.83a	2.94±0.48a	0.76±0.11a	0.74±0.29a	1.03±0.32a	0.26±0.16a	1.69±0.23a

Table S4. Results of Adonis tests for effects of forest types, N treatments, and their interactions on soil fungal communities.

	Df	Sums of squares	Mean squares	F	R^2	P
Forest type	4	8.68	2.17	10.51	0.506	**0.001**
N treat	2	0.45	0.23	1.09	0.026	0.303
Forest type×N treat	8	1.81	0.23	1.10	0.106	0.222
Residuals	30	6.20	0.21		0.361	
Total	44	17.14			1.000	

Table S5. Results of Adonis tests for effects of forest types, N treatments, and their interactions on soil fungal functional compositions.

	Df	Sums of squares	Mean squares	F	R^2	P
Forest type	4	1.27	0.32	8.19	0.427	**0.001**
N treat	2	0.15	0.07	1.89	0.049	0.067
Forest type×N treat	8	0.40	0.05	1.27	0.133	0.186
Residuals	30	1.17	0.04		0.391	
Total	44	2.99			1.000	

Table S6. Results of Adonis tests comparing pair-wise fungal community similarities derived in the matrix (Bray-Curtis distance) for each forest type and examining the significance of separation in different forest types. TMF: tropical montane rain forest, SEB: subtropical evergreen broadleaved forest, TDB: temperate deciduous broadleaved forest, TBCM: temperate broadleaved & conifer mixed forest, BF: boreal forest.

	TMF			SEB			TDB			TBCM			ALL		
	F	R^2	P	F	R^2	P	F	R^2	P	F	R^2	P	F	R^2	P
SEB	5.8	0.266	0.001												
TDB	10.9	0.406	0.001	8.3	0.341	0.001									
TBCM	11.6	0.420	0.001	8.2	0.338	0.001	9.1	0.363	0.001				10.3	0.506	0.001
BF	15.7	0.496	0.001	11.4	0.415	0.001	11.8	0.424	0.001	11.4	0.415	0.001			

Table S7. Results of Adonis tests comparing pair-wise fungal community similarities derived in the matrix (Bray-Curtis distance) for each N treatment and examining the significance of separation in different N treatments.

	CK			LN			ALL		
	F	R^2	P	F	R^2	P	F	R^2	P
LN	0.7	0.025	0.795				0.6	0.026	0.995
HN	0.5	0.018	0.986	0.5	0.016	0.996			

Table S8. Results of Adonis tests comparing pair-wise fungal functional composition similarities derived in the matrix (Bray-Curtis distance) for each forest type and examining the significance of separation in different forest types. TMF: tropical montane rain forest, SEB: subtropical evergreen broadleaved forest, TDB: temperate deciduous broadleaved forest, TBCM: temperate broadleaved & conifer mixed forest, BF: boreal forest.

	TMF			SEB			TDB			TBCM			ALL		
	F	R^2	P	F	R^2	P	F	R^2	P	F	R^2	P	F	R^2	P
SEB	17.4	0.521	0.001												
TDB	6.7	0.295	0.002	5.4	0.251	0.018									
TBCM	12.1	0.430	0.001	6.3	0.282	0.006	2.2	0.123	**0.097**				7.5	0.427	0.001
BF	11.5	0.419	0.001	11.1	0.410	0.001	4.1	0.204	0.004	4.9	0.233	0.002			

Table S9. Results of Adonis tests comparing pair-wise fungal functional composition similarities derived in the matrix (Bray-Curtis distance) for each N treatment and examining the significance of separation in different N treatments.

	CK			LN			ALL		
	F	R^2	P	F	R^2	P	F	R^2	P
LN	1.9	0.025	0.795				1.1	0.049	0.371
HN	0.9	0.018	0.986	0.5	0.016	0.996			
